# A prospective descriptive study on prevalence of catatonia and correlates in an acute mental health unit in Nelson Mandela Bay, South Africa

**DOI:** 10.1371/journal.pone.0264944

**Published:** 2022-03-08

**Authors:** Zukiswa Zingela, Louise Stroud, Johan Cronje, Max Fink, Stephan Van Wyk

**Affiliations:** 1 Nelson Mandela University, Gqeberha, South Africa; 2 Stony Brook University, New York, NY, United States of America; 3 Department of Psychiatry and Human Behavioural Sciences, Walter Sisulu University, Mthatha, South Africa; 4 Nelson Mandela Academic Hospital, Mthatha, South Africa; University of the West Indies at Saint Augustine, TRINIDAD AND TOBAGO

## Abstract

Catatonia is a psychomotor abnormality caused by neurological, medical or severe psychiatric disorders and substances. Its prevalence ranges from less than 10% to just above 60%. Diagnosis may be influenced by the screening tools used. Screening of new admissions to a mental health unit for catatonia was undertaken using three instruments to determine prevalence of catatonia. Participants ranged from age 16 years and over. Recruitment took place from September 2020 to August 2021. The setting was a mental health unit within a general hospital in Nelson Mandela Metro, South Africa. Five assessors were trained by the principal investigator to apply the Bush Francis Screening Instrument (BFCSI), the Bush Francis Catatonia Rating Scale (BFCRS), and the Diagnostic and Statistical Manual 5 (DSM-5), to assess participants. Clinical and demographic data were collected using a specially designed datasheet. Data analysis was performed to identify significant associations between presence or absence of catatonia and clinical and demographic data. Up to 241 participants were screened and 44 (18.3%) had catatonia. All 44 cases were identified through the BFCSI while the DSM-5 identified only 16 (6.6%%) of the 44 cases even though the remaining 28 (63.6%) participants still required treatment for catatonic symptoms. The DSM-5 diagnostic criteria excluded staring, which was the commonest sign of catatonia identified through the BFCSI [n = 33 (75%)]. Close to half (21; 47.7%) of those with catatonia on the BFCSI had schizophrenia. The rest had bipolar disorder (12; 27.3%), substance-induced psychotic disorder (7; 15.9%) and no specified diagnosis in one (1; 2.6%). The BFCSI was very effective at identifying catatonia while the DSM-5 was inadequate, missing close to 64% (28 of 44) of cases. Predictors of catatonia in this sample were a younger age and being male. A prevalence of 18.3%, indicates that assessment for catatonia should be routinely conducted in this and similar settings.

## Introduction

Catatonia manifests as a psychomotor abnormality presenting with disturbances in volition, motion and speech [1–8]. These abnormalities may occur as retarded movement (hypokinetic i.e., extremely slowed), excessive movement (hyperkinetic i.e., excited), disordered quality of movement (parakinesis) and/or as a mixture of these three abnormalities [1–2, 6–8]. There are no classical symptoms that are confined to any of these potential manifestations. The range and severity of symptoms and signs that may occur in catatonia have been well described by Bush et al, in their paper on standard examination and ratings scales for catatonia [7]. In severe cases, the motor abnormalities may occur with autonomic instability [2, 7–9].

There have been challenges in the recognition, diagnosis and treatment of catatonia in recent years [2, 9]. Reasons for these challenges include the assumption that catatonia is rare, the periodic and fluctuating nature of the presentation in some patients, the misinterpretation of catatonic as being “put on” by patients to gain attention and the waxing and waning of symptoms [2, 7, 9]. Wortzel et al tested knowledge of catatonia among a group of medical students, psychiatry residents and fellows and consultation liaison psychiatrists. They found that there were significant gaps in the understanding of catatonic features irrespective of stage of training [10].

Catatonia also has varying rates of prevalence in different populations, ranging from less than 10% to just above 60% [1–3]. The prevalence rates may be influenced by other factors, such as the recognition of catatonic signs, assessment tools used to screen for catatonia and the inter-rater reliability among clinicians assessing catatonia [6–13].

Despite some significant advances in the understanding of catatonia, there are still several important unanswered questions that emphasize the need to continue researching the subject and treatment thereof. These include the prevalence of catatonia in our setting and what the most effective management is given the resource challenges. This is because no studies on prevalence of catatonia in South Africa exist as yet. The importance of researching this is that knowledge gained could contribute towards defining the resource needs required to address catatonia in acute clinical settings. In this study, we provide data from a descriptive prevalence study in acute mental health unit, utilising the Bush Francis Catatonia Screening Instrument (BFCSI), Bush Francis Catatonia Rating Scale (BFCRS) and the Diagnostic and Statistical Manual-5 (DSM-5) as screening and assessment tools for catatonia.

### Aims

We aimed to investigate the prevalence of catatonia using the DSM-5 and BFCSI to screen for catatonia from September 2020 to August 2021, and to collect descriptive data on the clinical and demographic profile of patients who presented with catatonia. Demographic data collected included gender and age, while clinical information included diagnosis and substance use.

### Research design

This was a prospective, descriptive study that utilised a quantitative method.

## Materials and methods

Consenting participants were recruited from all new admissions by the lead researcher and five research assistants during the study period. We used the same definitions for catatonia as the screening tools i.e., two or more symptoms in the BFCSI and presence of three or more symptoms in the DSM-5 were indicative of catatonia. The BFCRS was used to assess for severity in those who screened positive for catatonia.

### Outline of the study process

The team of assessors included the principal investigator, two psychiatry residents, both with two years of experience in psychiatry, and three mental health professional nurses with a background of more than ten years each of working in mental health services.

Training for the five assessors on the use of the BFCSI/BFCRS and DSM-5 to assess catatonia was conducted by the principal investigator, focusing on description and definition of terms used in the tools, how to elicit the signs and how to capture the collected data on the data sheet. The research team alerted the treating doctor if there were any participants where catatonia was possibly missed. Any additional information collected during the assessment was also shared with the treating team to enable review of the patient’s diagnosis and treatment.

Evaluation of patients with catatonia by the treating team continued throughout admission while evaluation by the research team was done at the point of admission with the next evaluation conducted at one to three months after discharge.

### Setting

The study was conducted in a 35-bed acute mental health unit in Dora Nginza Hospital, which is in Nelson Mandela Bay Metro, Eastern Cape, Africa. This is a city with a population of 1.2 million people and has high rates of unemployment and morbidity of mental illness [13–15]. Mental health services at the MHU include 24-hour care for acute mental illness and electroconvulsive therapy. Referrals to the MHU come from other departments within the hospital as well as local clinics and district hospitals.

### Assessment tools

The BFCSI screening tool was used in this study to assess for catatonia and the BFCRS was used to assess for severity because they have been shown to have good interrater reliability in previous studies [1–3, 5, 7, 16]. The DSM 5 was included as an assessment tool due to its general use in undergraduate medical and postgraduate psychiatric training in South Africa. Assessors performed the ratings on consenting participants at the point of admission and for each tool (BFCSI, the BFCRS and DSM-5). Since interrater reliability may have an influence on the pick-up rate of screening instruments it may influence the reported prevalence rates for catatonia [5, 11, 16–18]. An investigation on interrater reliability (IRR) conducted on the first 10 cases with possible catatonia at the study site indicated a high interrater reliability for the BFCSI/BFCRS with a Krippendorff α = 0.798 and a low IRR for DSM-5 with a Krippendorff α = 0.565 [16].

### Sampling

Convenience sampling of all patients admitted to the MHU was done from September 2020 to August 2021. The number of patients that were expected to be admitted during the study period was approximately 250 based on admission figures for the unit over the previous six months. This was a lower number than usual due to the effects of the COVID-19 outbreak on admissions rates for non-COVID related admissions in the MHU and the rest of the hospital. This was mainly because of the need for social distancing that was required in the inpatient setting.

To determine the total sample size required for the prevalence study, the formula n = N/(1+Ne2) was utilised and yielded a minimum sample size of 153 subjects. A further 20% (30.6) was added to account for data entry errors and non-responses. The appropriate sample size of participants required to be screened for the prevalence of catatonia in the unit over 12 months was 184. Actual admissions during the study period were 270 and 241 (89.3%) of them gave consent for enrolment in the study.

### Participants

Most people admitted to the DNH MHU were involuntary admissions under the Mental Health Care Act of 2002 [19] from age 16 and older.

### Data management and analysis

The quantitative data collected on clinical and demographic parameters were summarised using descriptive statistics. The data are presented using frequency tables and percentages. The expected frequencies were calculated to determine the type of test best suited to determine the extent of any relative associations. Data analysis performed include the Chi-Squared Test and crosstabulations to determine significant associations between the presence or absence of catatonia and clinical and demographic information like age, sex, substance abuse, psychiatric diagnosis and co-occurring medical condition. The margin of error or confidence interval was set at 95%, with a standard deviation of 0.05. The Mann-Whitney U test was used to test whether there was a significant difference in screening tool scores (BFCSI, DSM-5) for age, sex, alcohol and cannabis use categories.

### Patient and public involvement

There was no patient and or public advisory committee that was set up for this research.

## Ethics and dissemination

The Human Research Ethics Committees at Walter Sisulu University, Nelson Mandela University, and the Eastern Cape Department of Health granted ethics approval while permission for data collection at the hospital was granted by the manager of the institution. We made participant information leaflets available in English or Xhosa and we issued these to all new admissions. We assessed capacity to consent on recruited participants based on the University of California Brief Assessment of Capacity to Consent, by designing a shortened version to decrease direct contact time with participants due to COVID-19 challenges [20, 21].

For participants who lacked capacity to consent, closest relatives were approached to provide proxy consent. This was aligned to the Helsinki Declaration and the National Department of Health Guidelines on ethics in health research [22–24]. Participants who lacked capacity to consent could therefore be included while still respecting their rights, enabling the inclusion of all participants or groups who could potentially benefit from scientific advances that could come about as a result of the research [23, 24]. Participant identification data were anonymised. All data related to the study was kept in a secure cupboard and stored on a password protected computer. Only the research team had access.

## Results

### Prevalence and demographic results

Of the 241 participants assessed, 197 (81.7%) did not have catatonia, while 44 (18.3%) screened positive for catatonia on the BFCSI and 16 (6.6%) were positive on the DSM-5. One of the 44 participants with catatonia on the BFCSI initially displayed echophenomena, with raised blood pressure on general examination and he had no history of pre-existing hypertension. This is one of the features of severity on the BFCRS and the participant was therefore monitored over a few hours post-admission due to concerns about episodic signs of catatonia. Significant rigidity was detected within a couple of hours after admission which meant the participant ended up with two signs on the BFCSI (echophenomena and rigidity) and a total BFCRS score of 3 in the presence of the abnormal blood pressure.

Most participants (159 [66%]) were between 16 and 35 years old with a mean age of 33.9 (SD = 12, 5) for the whole sample. In the group with catatonia 37 (15.3%) were in this age group with a mean age that was 2 years lower at 31.9 (SD = 11.5). In the total sample, males were 155 (64.3%) while females were 85 (35.3%) and one (0.4%) was of unknown sex. In the 44 with catatonia, males were 35 (79.6%) and females were 9 (20.5%). Up to 225 (93.4%) of the participants in the total sample were Black with 12 (5%) of Mixed Race, one (0.4%) White and one (0.4%) whose ethnic background was undefined. The prevalence rates of catatonia and the summary of the demographic profile are shown in Table 1.

**Table 1:** Prevalence rates of catatonia and demographic data.

Assessment tool	n (%) who screened positive	CI (%)
DSM-5	16 (6.6%)	4.2% - 10.7%
BFCSI	44 (18.6%)	14.1% - 23.8%
Demographics	**Participants without Catatonia**	**Participants with Catatonia**
Mean Age	33.9 years	31.9 years
Male	155 (64.3%)	35 (79.6%)
Female	85 (35.3%)	9 (20.5%)
Assessment tool	n (%)	CI (%)
DSM-5	16 (6.64%)	4.2% - 10.7%
BFCSI	44 (18.6%)	14.1% - 23.8%

### Assessment tools

In this sample, the BFCSI showed the highest rate of catatonia, with 44 (18.3.%) participants who screened positive and a confidence interval of 14.1% - 23.8%, followed by the DSM-5 which showed the lowest prevalence rate with 16 (6.6%) participants screening positive for catatonia and a confidence interval of 4.2–10.7%.

Of the 44 participants who were identified with catatonia, 16 (6.6%) were identified using both screening tools, 28 (11.6%) were on the BFCSI, with one (0.4%) of the 28 being the participant who initially presented with echophenomena alone. Seven (2.9%) of the participants who were missed when applying DSM-5 criteria, had a BFCSI score of two and still required treatment for catatonia while 21 (8.7%) had a score of more than two. All 28 (63.6%) cases missed by the DSM-5 still required treatment for catatonia, including the patient who initially presented with echophenomena and raised blood pressure. Table 2 shows the frequency of catatonia signs in this cohort.

**Table 2:** Frequency of catatonia signs in the sample (% of 44).

Signs of catatonia	Frequency
**Staring**	33 (75%)
**Mutism**	27 (61.3%)
**Rigidity**	18 (40.9%)
**Stupor**	16 (36.4%)
**Grimacing**	15 (34.1%)
**Posturing**	13 (29.6%)
**Participants with 2 signs of catatonia**	7 (15.9%)

### Psychiatric diagnosis

The most common DSM-5 psychiatric diagnosis (see Table 3) in the whole sample set was bipolar disorder (BD) with 86 (35.7%) participants, followed closely by schizophrenia with 71 (29.7%), then substance-induced psychotic (SIPD) disorder with 46 (19.1%) and Major Depressive Disorder (MDD) at 14 (5.9%) and substance induced bipolar disorder (SIBD) in 9 (3.7%) participants. The remaining 15 (6.2%) participants had a differential diagnosis of either BD or schizophrenia or had unknown or missing diagnoses. In the group of 44 with catatonia, 14 (31.8% of 44) had a mood disorder (12—BD and 2 –MDD), 21 (47.7%) had schizophrenia, and 9 (20.5%) had either a SIPD or SIBD.

**Table 3:** Psychiatric diagnoses.

Diagnoses		Total Sample n = 241	Patients with catatonia (n-44)	Average BFCRS Score
**Valid**	Bipolar Disorder	86 (35.7%)	12 (27.3%)	5
	Schizophrenia	71 (29.7%)	21 (47.7%)	5.6
	Substance Induced Psychotic Disorder	46 (19.1%)	7 (15.9%)	5.7
	Major Depressive Disorder	14 (5.8%)	2 (4.6%)	3.5
	Substance Induced Bipolar Disorder	9 (3.7%)	0	0
	Bipolar Disorder OR Schizophrenia	3 (1.2%)	0	0
	Missing or unknown	12 (5%)	2 (2.6%)	6
	Total	241	44	

In terms of BFCSI/BFCRS scores and diagnosis, as can be seen in Table 3, of the group with catatonia where the diagnosis was known, those with substances induced psychotic disorder were found to have the highest BFCRS scores, followed closely by those with schizophrenia and then bipolar disorder as shown.

### Substance use

Of the 241 participants, 163 (67.6%) had a history of substance use and 6 (2.3%) were unknown. Cannabis use was found in 106 (44%) of the total sample. Participants who were using cannabis accounted for 65% of the 163 participants with substance use. Up to 95 (28.6%) used alcohol, accounting for 58.3% of the 163 participants on substances. Up to 45 (18.7%) used unspecified substances, with 25 (10.4%) on amphetamines or methamphetamines, 21 (8.7%) on cocaine, and one (0.4%) on heroin. Up to 47 (19.5%) of the 163 participants who had used substances did so more than 4 times a week.

In the group of 44 with catatonia, 28 (63.6%) had substance use. Of the 28, up to 20 were on cannabis, which accounted for 45.5% of those which catatonia and substance use, while 13 (29.55%) were on alcohol. The rest were on methamphetamines, amphetamines or unspecified substances.

### Other medical illnesses

Co-occurring medical illness was present in 85 (35.3%) participants in the total sample, and 15 (6.2%) of them had catatonia. Up to 29 (12.0%) of the 85 indicated they had HIV and were on antiretroviral regimens with one (0.41%) of those having catatonia. There were however 35 (14.5%) who indicated they were on antiretroviral treatment for HIV, 9 (3.7%) who had viral count results for HIV and 28 (11.6%) who had CD4 count results on the online system when this was accessed for verification of blood results (with participant’s consent). Up to 7 (2.9%) of those with catatonia had CD4 count results and 4 (1.7%) of them had viral count results.

Other medical conditions reported were hypertension [8 (1.2%)], epilepsy [5 (2.1%)], tuberculosis [5 (2.1%)] and diabetes mellitus [5 (1.2%)]. Up to 110 (45.6%) had TSH results available with 27 (11%) of them those with catatonia and in both groups most of the results fell within the normal range. Table 4 shows a summary of clinical data.

**Table 4:** Clinical data for participants with and without catatonia.

History of medical Illness: catatonia vs no catatonia participants (% of n—241)				Substances (n = 163)	
	Medical Illness	HIV	CD4 Count	Cannabis	Alcohol
**Catatonia**	15 (6.2%)	1 (0.4%)	7 (2.9%)	20 (8.3%)	13 (5.4%)
**No catatonia**	70 (29.1%)	6 (2.5%)	21 (8.7%)	86 (35.7%)	82 (35.4%)
**Total**	85 (35.3%)	29 (2.9%)	28 (11.6%)	106 (44%)	95 (39.4%)

### Course of catatonia in this sample

At the time of concluding data collection, 14 (5.8% of 241 and 31.8% of 44) of the 44 participants with catatonia on the BFCSI were still in hospital, three (1.2% of 241 and 6.8% of 44) had been transferred to the local psychiatric hospital and 30 (12.5% of 241 and 68.2% of 44) had been discharged home.

There were no fatalities in the 44 participants with catatonia during the study period and only one (0.4% of 241 and 2.3% of 44) participant had a positive BFCSI one month after discharge for which he was readmitted.

## Data analysis results

### Chi-squared test

A chi-squared test of association was performed to determine whether there was a statistically significant association between catatonia (positive screen on the BFCSI) and diagnoses. For BFCSI and diagnoses, there were no statistically significant associations (*χ*^2^(6) = 10.164, *sig* = 0.113).

### Crosstabulations

A number of crosstabulation were performed. This included determining whether there was a statistically significant association between a positive BFCSI and age category. It was found that there was a significant association between a positive BFCSI and age (*χ*^2^ = 17.393, *df* = 3, *sig* = 0.001, *Cramer′s V* = 0.269). The crosstabulation indicated that more patients diagnosed with catatonia were in the age category 16–35 years compared to those in age category 36–65 years.

A crosstabulation was also performed to determine whether there was a statistically significant association between catatonia (based on positive the BFCSI) and substance use. No statistically significant association was obtained (*χ*^2^(1) = 0.079, *sig* = 0.779).

In addition, a crosstabulation was performed to determine whether there was a statistically significant association between catatonia (based on the BFCSI) and co-occurring medical illness, specifically Human Immunodeficiency Virus (HIV) infection and CD4 count. Regarding HIV, no statistically significant association was obtained (*χ*^2^(2) = 0.265, *sig* = 0.876). Regarding CD4 count however, a statistically significant association was obtained (*χ*^2^(2) = 16.244, *sig* = 0.000). The results indicate that in this sample, there was a greater likelihood of patients diagnosed with catatonia having a CD4 count of 500 or less.

Other crosstabulations which were analysed for significant associations were between catatonia and alcohol use, and catatonia and cannabis use, but these also did not yield any significant findings.

#### The Mann-Whitney U Test

A Mann-Whitney U test was used to determine whether there was a statistically significant difference in the BFCSI score dependent on the sex of the patient, the age, alcohol and cannabis use. A statistically significant difference was obtained for the average BFCSI score according to the sex of patient (U = 5699.5, Z = -1.962, sig = 0.05) with male patients obtaining the higher BFCSI mean rank compared to the female patients (Mean Ranks (MR)(Male) = 124.75 and MR(Female) = 110.05, respectively). This indicates that the male patients had on average, a higher BFCSI score when compared to the female patients.

Also, a statistically significant difference was obtained for the average BFCSI score according to the age category of patients (U = 5637.5, Z = -2.048, sig = 0.041) with the patients in the age category 35 years or younger obtaining the higher BFCSI mean rank compared to the patients in the age category older than 35 years (MR(<35) = 126.27 and MR(>35) = 110.6, respectively). This indicates that younger patients had on average, a higher BFCSI score when compared to the older patients.

### Logistic regression

A minimum recommended sample size of 500 is required to perform a logistic regression in the data analysis of observational studies in large populations [25]. Due to the fact that the sample size was 241 in this sample, we have not included the findings of this type of data analysis in the final results section.

### Summary of significant clinical and demographic correlations

Analysis of all the data indicates that there was a greater probability of a person screening positive for catatonia on the BFCSI if they were younger than 35 and male and they also tended to have a higher BFCSI score on average compared to patients older than 35 or female patients, or put another way, BFCSI and DSM-5 scores increased with decreasing age.

## Discussion

The 12-month prevalence rate of catatonia in this cohort from an acute mental health unit within a general hospital, was 18.3%. This rate falls within the range reported in other studies [2, 3, 5, 26] and highlights the importance of assessing patients for catatonia in such settings so that early and targeted multidisciplinary treatment can be initiated.

Many studies have identified various causes of catatonia with schizophrenia and affective disorders still being the most common psychiatric diagnoses that underlie the presentation of catatonia [23–24, 26–30]. The predominant diagnosis in those with catatonia in this sample was schizophrenia in close to 48% followed by bipolar disorder in 32%. This means schizophrenia and bipolar disorder accounted for 80% of the underlying psychiatric causes in this cohort. These findings are consistent with the results of previous studies [23–24, 26–33].

The DSM-5 is a useful diagnostic tool in the clinical setting; however, when it was used to assess catatonia in our study, 63.6% of cases with catatonia who required treatment were missed. This means that patients who were deemed sick enough to require inpatient intervention for their catatonic presentation might not have received the necessary treatment to adequately address their condition if the clinicians at the study site relied on DSM-5 only to assess for catatonia.

As the findings in our study suggest, the DSM-5 diagnostic criteria fell significantly short in this sample set, missing close to 64% of cases with catatonia. We identified staring as the most frequently occurring sign, seen in 75% of the 44 cases. This is similar to the study by Yitayih et al., where staring occurred in 88.9% of 18 patients with catatonia [12]. Considering that staring does not feature as one of the signs listed in the DSM-5 diagnostic criteria for catatonia, this may be one of the deficits that may account for the lower pick-up rate of catatonia when applying DSM-5 diagnostic criteria. DSM-5 does include rigidity in the diagnostic criteria for catatonia, but in its extreme form i.e., when it presents as catalepsy and posturing. The BFCSI on the other hand allows for degrees of rigidity, which again might account for the wide margin between the pick-up rate of catatonia when comparing the two tools. In this sample, rigidity was seen in 18 (40.9%) participants. Wortzel et al suggest that another contributing factor for underdiagnosis of catatonia could be related to clinicians’ inadequate understanding of catatonia and its heterogeneous presentations [34]. This may be another factor that could contribute to these differences when applying these tools to assess for catatonia in the clinical setting.

Significant clinical and demographic factors associated with catatonia in our study included a younger age and being male. Rogers et al indicated similar findings in a recent study on the clinical and demographic profile of participants with catatonia. In their study, catatonia was also associated with a younger age and Black ethnicity [35]. Although the majority of those who presented with catatonia were Black in our study, there were too few participants from other ethnic groups to make meaningful comparisons.

## Study strengths and limitations

This is the first study to provide data and findings on prevalence of catatonia in South Africa. This can therefore lay the groundwork for further studies in catatonia in this and similar settings. The study also identifies possible predictors of catatonia which may be useful to clinicians for assessment and decision making purposes.

The lower admission rates due to the COVID-19 pandemic affected the limited number of participants with catatonia recruited in this sample. This may have implications for generalizability of the study results. Screening for catatonia and assessment of severity was done using tools which have not been validated for use in South Africa. Nonetheless, the BFCSI/BFCRS and DSM-5 are still used extensively by clinicians in South Africa to assess for catatonia. The screening tools we used in this study excluded the ICD-10 and Braunig Catatonia Rating Scale, thus limiting the range of comparison that could be performed between rating scales. The assessment for catatonia was done at the point of admission and did not include those who might have gone on to develop catatonia during the rest of the inpatient period.

## Conclusion

The findings of this study indicate that the prevalence of catatonia is high enough to warrant routine screening of all admissions to acute mental health units and similar settings in South Africa. The DSM-5 has notable deficits which limit its utility in the assessment of catatonia in the clinical setting and if used as the sole means of assessing patients for catatonia, it may result in missing seriously ill patients with catatonia who require treatment. These study findings are important for all settings where clinicians may come across patients with catatonia, which may include not only mental health units and psychiatric hospitals but also departments of neurology, internal medicine and accident and emergency settings.

## Supporting information

S1 Data (XLSX)
